# Comparison of Clinical and Physiological Parameters for Benign Prostatic Hyperplasia in Hypertensive and Normotensive Patients

**DOI:** 10.3389/fphys.2018.01330

**Published:** 2018-09-24

**Authors:** Xian-Tao Zeng, Hong Weng, Jing Xiong, Qiao Huang, Lin-Lu Ma, Ying-Hui Jin, Xing-Huan Wang

**Affiliations:** ^1^Department of Urology, Zhongnan Hospital of Wuhan University, Wuhan, China; ^2^Center for Evidence-Based and Translational Medicine, Zhongnan Hospital of Wuhan University, Department of Evidence-Based Medicine and Clinical Epidemiology, The Second Clinical College of Wuhan University, Wuhan, China; ^3^Department of Cardiology, The First Affiliated Hospital of Henan University of Traditional Chinese Medicine, Zhengzhou, China

**Keywords:** hypertension, benign prostatic hyperplasia, prostate volume, prostate specific antigen, international prostate symptom score, body mass index, fasting blood sugar

## Abstract

**Objective:** To discover the correlation of clinical and physiological measures for benign prostatic hyperplasia in hypertensive and normotensive patients.

**Methods:** From September 2016 to October 2017, 435 patients were enrolled for further selection. The parameters evaluated for eligible patients included prostate volume, systolic blood pressure, diastolic blood pressure, international prostate symptom score, etc. Then the eligible patients were divided into two groups according to hypertension condition, and the clinical and physiological parameters were compared between two groups. The Pearson’s correlation coefficient was used to test the linearity of the relationships of these clinical and physiological components with prostate volume, total prostate specific antigen, and international prostate symptom score.

**Results:** Finally, 350 patients were involved in this study, including 117 with hypertension and 233 without hypertension. Weight, body mass index, systolic blood pressure, and diastolic blood pressure were significantly different between the hypertension and normotension groups. In the normotension group, there were positive correlations between weight, body mass index, age, and prostate volume; between fasting blood sugar, systolic blood pressure, diastolic blood pressure, and total prostate specific antigen; between fasting blood sugar and international prostate symptom score. In the hypertension group, there were positive correlations between age and total prostate specific antigen and international prostate symptom score; between weight and prostate volume; between systolic blood pressure and total prostate specific antigen.

**Conclusion:** This study indicated that there might be no significant association between hypertension and prostate volume.

## Introduction

Epidemiological studies have shown that multiple factors may increase the risk of benign prostatic hyperplasia (BPH), such as advanced age, obesity, increased free prostate specific antigen (PSA) level, cardiovascular disease (CVD), and the use of β-blocker medications (Meigs et al., 2001; Kok et al., 2009). It is known that hypertension is an independent risk factor for CVD (D’Agostino et al., 2017), and hypertension and BPH are two common disorders in aged men. Besides, emerging evidence indicates that both BPH and hypertension are components of metabolic syndrome (MetS) (Corona et al., 2014). As the rapid aging of population progress, BPH, and hypertension have become a significant public health problem worldwide. Approximately 30% of men with BPH suffer from hypertension simultaneously (Mathur et al., 2014). In 1966, a retrospective study was performed to investigate the association among BPH, hypertension, and T2DM (Bourke and Griffin, 1966). Then in 1995, a prospective study suggested that hypertension was correlated with the increased odds of surgery for BPH (Gann et al., 1995). However, because the prevalence of hypertension and BPH both increase with age, the relationship between these two conditions still remains unclear, especially in terms of the effectiveness and safety of surgery. The aim of this study was to evaluate the BPH components in hypertensive and normotensive patients who underwent surgery for BPH.

## Materials and Methods

### Study Design and Subjects

The study subjects were selected from the Bladder Cancer and Benign Prostatic Hyperplasia Study in Chinese Population (BPSC), which was a prospective study investigating the clinical effect of interventional therapy and the risk factors for bladder cancer and BPH (Zeng et al., 2018). This research was reviewed and approved by the Committee for Ethical Affairs of the Zhongnan Hospital of Wuhan University at Wuhan City, Hubei Province (Approval No. 2016028). All participants were Chinese and signed the informed consent before enrollment. From September 2016 to October 2017, 435 patients enrolled in the BPSC database were included for selection. Patients who were diagnosed with BPH, underwent surgery for this disorder, and whose data free of missingness were included in this research. Patients with prostate cancer, bacteriuria, pyuria, or previous surgery involving prostate and urethral were excluded from the study. All eligible patients were finally classified into two groups according to the status of hypertension, i.e., the hypertensive and normotensive group; and the hypertensive group was further stratified into three subgroups based on the levels of hypertension. The present study was conducted and reported in accordance with the STrengthening the Reporting of OBservational studies in Epidemiology (STROBE) statement (Vandenbroucke et al., 2014).

### Measurements and Data

Detailed medical history and physical examination were obtained from all included patients. The weight and height of each participant were measured, and body mass index (BMI) was calculated as weight in kilograms divided by height in meters squared (kg/m^2^). The symptoms of the patients were assessed using international prostate symptom score (IPSS). Hypertension was diagnosed according to the systolic blood pressure (SBP; mmHg) and diastolic blood pressure (DBP; mmHg). Fasting blood samples were drawn from patients to determine fasting blood sugar (FBS, ng/mL), total prostate specific antigen (t-PSA; ng/mL), serum K^+^ (mmol/L), serum Na^+^ (mmol/L), serum Cl^−^ (mmol/L), serum Ca^2+^ (mmol/L), and serum Mg^2+^ (mmol/L). Prostate volume (PV) was measured based on the prostate ellipsoid formula, by multiplying the largest anteroposterior (height, H), transverse (width, W), and cephalocaudal (length, L) prostate diameters using B-ultrasonography (transrectal or transabdominal) and then calculating using the ellipsoid formula {PV = π/6 × [H (cm) × W (cm) × L (cm)]} (Terris and Stamey, 1991).

### Statistical Analysis

All analyses were carried out using the SPSS 19.0 software. We compared age, weight, height, BMI, FBS, IPSS, t-PSA, PV, SBP, DBP, serum K^+^, serum Na^+^, serum Cl^−^, serum Ca^2+^, and serum Mg^2+^ between the hypertensive and normotensive group, and then compared these parameters among the three subgroups with different levels of hypertension. The Student’s t-test was used to describe the difference of above parameters between the hypertensive group and the normotensive group. The Pearson’s correlation analysis was applied to examine linear relationships between these clinical and physiological components and PV, t-PSA, and IPSS. A *p* < 0.05 was considered statistically significant.

## Results

A total of 350 patients were included in this study, among whom 117 patients with hypertension, including 80 grade 1, 30 grade 2, and 7 grade 3 hypertension cases. Baseline characteristics of all these patients were presented in **Table 1**. The mean age of the patients was 70.943 ± 7.362 years. The mean ages for the two groups were respectively 70.657 ± 7.327 years and 71.513 ± 7.431 years, and there was no statistically difference (*p* = 0.305). Weight, BMI, SBP, and DBP were significantly lower in the hypertensive group (*p* < 0.05). No significant difference was detected between the two groups in terms of all the other variables.

**Table 1:** Baseline characteristics of benign prostatic hyperplasia patients with and without hypertension.

Characteristic	Total	Patients with hypertension	Patients without hypertension	*p*
No of patients	350	117	233	NA
Age (years)	70.943 ± 7.362	70.657 ± 7.327	71.513 ± 7.431	0.305
Height (cm)	167.490 ± 5.581	167.316 ± 5.469	167.835 ± 5.807	0.417
Weight (kg)	65.453 ± 9.952	64.493 ± 9.786	67.350 ± 10.046	0.012*
BMI (kg/m^2^)	23.363 ± 3.381	23.068 ± 3.401	23.940 ± 3.279	0.024*
FBS (ng/mL)	80.668 ± 44.414	83.759 ± 45.413	74.402 ± 41.821	0.073
t-PSA (ng/mL)	5.190 ± 8.347	4.922 ± 6.673	5.703 ± 10.871	0.485
IPSS	23.329 ± 6.470	23.262 ± 6.609	23.460 ± 6.216	0.792
PV (mL)	63.084 ± 41.059	62.932 ± 37.882	63.389 ± 46.972	0.928
Serum K^+^	3.917 ± 0.375	3.910 ± 0.365	3.929 ± 0.396	0.656
Serum Na^+^	140.876 ± 8.262	140.438 ± 9.845	141.725 ± 3.458	0.08
Serum Cl^−^	104.831 ± 6.517	104.677 ± 7.639	105.133 ± 3.391	0.45
Serum Ca^2+^	2.191 ± 0.176	2.185 ± 0.185	2.204 ± 0.158	0.342
Serum Mg^2+^	0.870 ± 0.122	0.871 ± 0.128	0.867 ± 0.108	0.811
SBP	132.420 ± 17.159	123.575 ± 9.793	150.034 ± 14.964	<0.00*
DBP	79.211 ± 11.163	75.137 ± 8.163	87.325 ± 11.913	<0.00*

*^∗^statistically significant. BMI, body mass index; FBS, fasting blood sugar; t-PAS, total prostate specific antigen; IPSS, international prostate symptom score; PV, prostate volume; SBP, systolic blood pressure; DBP, diastolic blood pressure; NA, Not Applicable.*

**Table 2** demonstrated the association between the parameters of hypertensive patients including PV, t-PSA, and IPSS and the other parameters of all these patients. In the analysis, a positive link was established between PV and weight which indicated that patients with higher weight were likely to have larger PV (*r* = 0.198, *p* = 0.034). A negative correlation was found between FBS and PV (*r* = −0.206, *p* = 0.034). Two parameters showing a significant link with t-PSA were age (*r* = 0.211, *p* = 0.024) and SBP (*r* = 0.231, *p* = 0.013). Similarly, age was positively associated with IPSS (*r* = 0.206, *p* = 0.029); whereas serum Ca^2+^ was negatively correlated with IPSS (*r* = −0.285, *p* = 0.003).

**Table 2:** Comparison of PV, t-PSA, and IPSS of 117 hypertensive patients with other parameters.

Patients with Hypertension	PV (mL)		t-PSA (ng/mL)		IPSS	
	r	*P*	r	*P*	r	*P*
Age (years)	−0.024	0.795	0.211	0.024*	0.206	0.029*
Height (cm)	0.103	0.276	−0.093	0.33	0.004	0.963
Weight (kg)	0.198	0.034*	−0.118	0.214	−0.128	0.178
BMI (kg/m^2^)	0.156	0.097	−0.084	0.378	−0.151	0.113
FBS (ng/mL)	−0.206	0.034*	0.092	0.352	0.038	0.699
Serum K^+^	−0.086	0.365	−0.16	0.093	−0.111	0.245
Serum Na^+^	−0.049	0.606	−0.05	0.603	0.074	0.442
Serum Cl^−^	−0.094	0.321	−0.16	0.093	0.039	0.685
Serum Ca^2+^	<0.001	0.998	−0.015	0.876	−0.285	0.003*
Serum Mg^2+^	−0.074	0.542	−0.155	0.199	−0.06	0.626
SBP	−0.054	0.565	0.231	0.013*	0.149	0.116
DBP	−0.046	0.621	−0.035	0.715	−0.014	0.881
PV	1	NA	0.139	0.139	0.076	0.424
t-PSA	0.139	0.139	1	NA	−0.031	0.75
IPSS	0.076	0.424	−0.031	0.75	1	NA

*^∗^statistically significant; −, negative correlation; BMI, body mass index; FBS, fasting blood sugar; t-PAS, total prostate specific antigen; IPSS, international prostate symptom score; PV, prostate volume; SBP, systolic blood pressure; DBP, diastolic blood pressure; NA, Not Applicable.*

According to the stratified analysis by grade of hypertension, we found that weight (*r* = 0.232, *p* = 0.041) and age (*r* = 0.408, *p* = 0.025) were significantly correlated with PV in grade 1 and grade 2 patients, respectively; age (*r* = 0.274, *p* = 0.016), FBS (*r* = 0.314, *p* = 0.008), and PV (*r* = 0.328, *p* = 0.004) were significantly related to t-PSA in grade 1 level patients; negative correlation were also observed between serum Cl^−^ (*r* = −0.302, *p* = 0.008), weight (*r* = −0.423, *p* = 0.02) and t-PSA levels in grade 1 level and grade 2 level, respectively. In addition, serum Ca^2+^ was negatively correlated with IPSS (*r* = −0.341, *p* = 0.003) in grade 2 patients. **Table 3** presented the results of stratified analysis.

**Table 3:** Comparison of PV, t-PSA, and IPSS with other parameters across three hypertensive groups.

Patients with Hypertension	PV (mL)		t-PSA (ng/mL)		IPSS	
	r	*P*	r	*P*	r	*P*
**Grade 1 level (80 patients)**						
Age (years)	−0.185	0.103	0.274	0.016*	0.214	0.06
Height (cm)	0.132	0.253	0.136	0.245	−0.061	0.598
Weight (kg)	0.232	0.041*	0.088	0.452	−0.137	0.234
BMI (kg/m^2^)	0.186	0.106	0.023	0.843	−0.131	0.259
FBS (ng/mL)	−0.228	0.052	0.314	0.008*	0.169	0.156
Serum K^+^	−0.118	0.302	−0.068	0.562	−0.113	0.33
Serum Na^+^	−0.065	0.572	0.03	0.797	0.134	0.245
Serum Cl^−^	−0.123	0.287	−0.302	0.008*	0.026	0.821
Serum Ca^2+^	0.026	0.825	−0.043	0.712	−0.341	0.003*
Serum Mg^2+^	0.039	0.793	−0.208	0.162	−0.169	0.251
SBP	−0.065	0.567	0.03	0.793	0.089	0.437
DBP	0.054	0.639	−0.017	0.884	−0.161	0.159
PV	1	NA	0.328	0.004*	0.089	0.441
t-PSA	0.328	0.004*	1	NA	0.059	0.615
IPSS	0.089	0.441	0.059	0.615	1	NA
**Grade 2 level (30 patients)**						
Age (years)	0.408	0.025*	0.124	0.513	0.27	0.164
Height (cm)	0.004	0.981	−0.227	0.227	0.267	0.17
Weight (kg)	0.093	0.625	−0.423	0.02*	−0.086	0.663
BMI (kg/m^2^)	0.094	0.622	−0.305	0.101	−0.244	0.211
FBS (ng/mL)	−0.115	0.569	<0.001	1.000	−0.35	0.087
Serum K^+^	0.056	0.772	−0.156	0.42	−0.329	0.094
Serum Na^+^	−0.04	0.837	−0.164	0.397	0.041	0.839
Serum Cl^−^	−0.037	0.847	−0.344	0.068	0.192	0.338
Serum Ca^2+^	−0.148	0.453	0.067	0.735	−0.154	0.454
Serum Mg^2+^	−0.222	0.335	−0.204	0.375	0.132	0.59
SBP	0.162	0.393	0.3	0.107	0.23	0.238
DBP	−0.287	0.124	−0.277	0.138	−0.129	0.514
PV	1	NA	0.345	0.062	0.097	0.624
t-PSA	0.345	0.062	1	NA	−0.035	0.858
IPSS	0.097	0.624	−0.035	0.858	1	NA
**Grade 3 level (7 patients)**						
Age (years)	0.237	0.608	0.666	0.102	−0.176	0.706
Height (cm)	−0.143	0.759	−0.078	0.868	−0.308	0.501
Weight (kg)	−0.042	0.929	−0.217	0.641	−0.116	0.804
BMI (kg/m^2^)	0.109	0.816	−0.34	0.456	0.114	0.808
FBS (ng/mL)	−0.507	0.245	0.095	0.839	−0.507	0.245
Serum K^+^	−0.558	0.193	−0.346	0.448	0.64	0.122
Serum Na ^+^	0.369	0.415	−0.006	0.99	−0.568	0.184
Serum Cl^−^	−0.034	0.943	0.299	0.515	−0.179	0.701
Serum Ca^2+^	−0.178	0.702	−0.022	0.963	0.28	0.543
Serum Mg^2+^	NA	NA	NA	NA	NA	NA
SBP	0.384	0.395	−0.011	0.981	−0.026	0.956
DBP	0.305	0.506	−0.55	0.201	0.081	0.863
PV	1	NA	−0.186	0.69	0.012	0.979
t-PSA	−0.186	0.69	1	NA	−0.463	0.295
IPSS	0.012	0.979	−0.463	0.295	1	NA

*^∗^statistically significant; − negative correlation. BMI, body mass index; FBS, fasting blood sugar; t-PAS, total prostate specific antigen; IPSS, international prostate symptom score; PV, prostate volume; SBP, systolic blood pressure; DBP, diastolic blood pressure; NA, Not Applicable.*

The same comparison between the parameters of normotension patients were shown in **Table 4**. Age was still significantly correlated with PV (*r* = 0.186, *p* = 0.004), like in grade 2 hypertension. Besides, both weight and BMI were associated with PV (*p* = 0.046 and *p* = 0.011 respectively). A negative association was established between serum Cl^−^ and PV (*r* = −0.146, *p* = 0.03). There were also positive correlations between FBS, SBP, DBP and t-PSA levels (*p* = 0.005, *p* = 0.036, and *p* = 0.007, respectively). Moreover, FBS was positively related to IPSS (*r* = −0.172, *p* = 0.011), while height was negatively correlated with IPSS (*r* = 0.263, *p* < 0.001).

**Table 4:** Comparison of PV, t-PSA, and IPSS of 233 normotensive patients with other parameters.

Patients without Hypertension	PV (mL)		t-PSA (ng/mL)		IPSS	
	r	*P*	r	*P*	r	*P*
Age (y)	0.186	0.004*	−0.076	0.264	0.057	0.401
Height (cm)	−0.081	0.222	0.102	0.139	−0.172	0.011*
Weight (kg)	0.132	0.046*	−0.052	0.449	−0.07	0.301
BMI (kg/m^2^)	0.169	0.011*	−0.107	0.124	−0.007	0.917
FBS (ng/mL)	−0.031	0.652	0.193	0.005*	0.263	<0.001*
Serum K^+^	−0.009	0.895	−0.029	0.677	<0.001	0.994
Serum Na^+^	0.019	0.782	−0.02	0.773	−0.051	0.462
Serum Cl^−^	−0.146	0.03*	0.01	0.889	0.089	0.199
Serum Ca^2+^	−0.069	0.309	−0.107	0.124	−0.042	0.543
Serum Mg^2+^	−0.081	0.295	−0.127	0.107	−0.112	0.159
SBP	−0.04	0.542	0.142	0.036*	−0.124	0.067
DBP	−0.006	0.933	0.181	0.007*	−0.102	0.13
PV	1	NA	0.062	0.365	0.099	0.143
t-PSA	0.062	0.365	1	NA	0.092	0.188
IPSS	0.099	0.143	0.092	0.188	1	NA

*^∗^statistically significant; −, negative correlation; BMI, body mass index; FBS, fasting blood sugar; t-PAS, total prostate specific antigen; IPSS, international prostate symptom score; PV, prostate volume; SBP, systolic blood pressure; DBP, diastolic blood pressure; NA, Not Applicable.*

## Discussion

In this study, clinical and physiological parameters of BPH were compared between hypertension and normotension patients undergoing surgery. As a result, significant correlations were observed between FBS, weight and PV; between age, SBP and t-PSA level; between age, serum Ca^2+^ and IPSS in BPH patients with hypertension. In the BPH patients without hypertension, age, weight, BMI, and serum Cl^−^ were notably correlated with PV; FBS, SBP, and DBP were significantly associated with t-PSA level; height and FBS were remarkably linked to IPSS.

The connection of hypertension and BPH has been studied for more than 50 years since Bourke JB and Griffin JP suggested an association between hypertension and BPH etiology for the first time (Bourke and Griffin, 1966). However, the pathogenesis of BPH is still unclear, which probably depends on a series of complex mechanism. It has attracted the attention of more researchers. Hypertension seems to play a certain role in pathogenesis of BPH via both static and dynamic components. Numerous studies also proved that patients with hypertension had a higher prevalence of lower urinary tract symptoms (LUTS) (Joseph et al., 2003; Rohrmann et al., 2005; Seim et al., 2005; Wang et al., 2015). In 2003, Joseph et al. (2003) performed a study to investigate the risk factors of BPH-related urinary symptoms, and found that the African-American men with history of hypertension had 1.76 times the risk of moderate to severe LUTS (*OR* = 1.76, 95%CI = 1.26–2.45) and moderate to severe obstructive symptoms (*OR* = 1.76, 95%CI = 1.20–2.58), and a more than twofold increase in the risk of moderate to severe irritative symptoms (*OR* = 2.10, 95%CI = 1.54–2.86); after adjustment for lifestyle risk factors and medical conditions, the associations remained significant. Then the Third National Health and Nutrition Examination Survey (NHANES III) suggested that men with a history of hypertension had a significantly higher risk of BPH-related LUTS than those who never had this disease (*OR* = 1.76, 95% CI = 1.20–2.59) (Rohrmann et al., 2005). Nevertheless, the study by Wang et al. (2015) revealed a non-significant association between history of hypertension and BPH-related LUTS in Chinese population.

As we know, BPH patients with hypertension who underwent surgery for BPH would have an increased surgical risk, especially those having bleeding during intraoperative and postoperative. The Physician’s Health Study indicated that hypertension was associated with the increased odds of surgery for BPH (Gann et al., 1995). Hence, we compared the physiological parameters of BPH which might be associated with hypertension. Considering serum K^+^ levels outside the interval of 4.1–4.7 mmol/L were associated with increased mortality risk in patients with hypertension (Krogager et al., 2017); BPH patients with higher serum K^+^ levels might face higher surgical risk, especially those with hypertension patients. Our results suggested that the serum K^+^ levels between hypertension group and normotension group were similar. Evidences showed that serum Cl^−^ was an independent predictor of mortality in hypertensive patients (McCallum et al., 2013). Our results indicated that serum Cl^−^ was negatively correlated with t-PSA in grade 1 level hypertensive patients and with PV in normotension patients. Additionally, some researches indicated that hypertensive patients had an impaired intracellular ion content, characterized by decreased intracellular Mg^2+^ as well as increased intracellular Ca^2+^ and Na^+^ compared to healthy controls (Touyz et al., 1992), the inverse correlation of serum Mg^2+^ levels with SBP and DBP were observed (Ma et al., 1995). Our results revealed that only serum Ca^2+^ was related to IPSS in the overall hypertension group and grade 1 subgroup. The above results indicated that it was required to pay attention to the serum Ca^2+^ level in hypertensive patients undergoing surgery for BPH.

BMI has been considered as a risk factor for BPH, diabetes and hypertension (Decoda Study et al., 2008). Some publications support a positive correlation between PV and FBS in non-diabetic BPH patients (Kim et al., 2011; Ozcan et al., 2017). MetS is a well-recognized cluster of cardiovascular (CV) risk factors including obesity, hypertension, dyslipidemia, and hyperglycaemia (Hammarsten et al., 1998). Emerging evidence indicates that BPH and its related LUTS represent other clinical conditions frequently observed in subjects with MetS (Hammarsten et al., 1998; Corona et al., 2014). We also compared height, weight, BMI, and FBS in this study. The results showed that weight and BMI were significantly different between hypertension group and normotension group. Besides, there was a significant correlation between weight and PV in the hypertension group, grade 1 level hypertension group, and normotension group.

FBS was positively associated with t-PSA and IPSS in normotension group; whereas it showed a negative association with PV in hypertension group. When we stratified hypertension patients, we found FBS was positive link to grade 1 level (*r* = 0.314, *p* = 0.008) but not linked to grade 2 level (*r* < 0.001, *p* = 1.000). FBS is the indicator of new-onset DM and one of the indicators of MetS. Hypertension is closely related to DM, with approximately 50% of patients who have hypertension developing hyperinsulinemia and 75% of patients who have T2DM developing hypertension (Abuissa et al., 2005). New-onset DM refers to forms of T2DM that develop during the therapeutic processes of other diseases such as hypertension (Li et al., 2017), this might explain why the FBS was associated to grade 1 level hypertensive patients in present study. Moreover, DM is known to be associated with an increased risk of greater severity of BPH (Boon et al., 2001). Our results indicated there was no difference of FBS between hypertensive and normotensive group; however, it is negative linked to PV in hypertensive patients. The reason might be that FBS is more sensitivity to grade 1 lever hypertension than grade 2 and 3 level hypertension. In addition, this might mean that BPH patients with grade 1 level hypertension are more susceptible to impaired FBS, which reminded urologist, cardiologist, and endocrinologist that more attention should be paid to the patients whom undergo BPH with hypertension.

To our knowledge, this is the first study comparing clinical and physiological parameters of BPH in hypertensive and normotensive patients. The major limitation of the present study was the relatively small sample size, which might influence the accuracy of results, especially when there were only 7 patients with grade 3 level group after stratifying the BPH patients according to hypertension. Interestingly, none of targeted parameters were found to have positive or negative associations in this group, this probably due to too small samples. Hence, we suggest to perform large-scale studies in the future to further verify our results, especially the meta-analysis (Zeng et al., 2015) when relevant studies are enough. Moreover, we could only perform this cross-sectional study due to the insufficient data. Future studies with the prospective cohort design are needed to clarify if there exists a positive correlation between PV and hypertension in BPH patients. The influence of effectiveness and safety of surgery owing to BPH with hypertension is also a valuable topic which is well worth of research. Of course, the mechanisms of the positive and negative correlations in our study are also necessary to be further explored in the future. There are many mechanisms proposed to associate the development of BPH in hypertensive patients. Firstly, FBS represented the level of MetS and degree of DM, approximately 50% of patients who have hypertension developing hyperinsulinemia and 75% of patients who have T2DM developing hypertension (Abuissa et al., 2005); hence, hyperinsulinemia associated with increased sympathetic activity via enhanced glucose metabolism in ventromedial hypothalamic neurons (Landsberg, 1986) may contribute to an increase in the activation of the alpha adrenergic pathway with contraction of smooth muscle of the urinary tract contributing to the development of BPH. Moreover, weight was associated with increased risk of BPH and hypertension, weight combined with hypertension would resulted in regression of the gland volume via abnormal adipokine levels (such as leptin vs. adiponectin ratio) (Ding et al., 2017).

## Conclusion

In conclusion, current study suggested that weight and BMI were different in the hypertensive and normotensive BPH patients, which were both correlated with PV in non-hypertension group; only weight was associated with PV in hypertension group. Age was positively correlated with PV in normotension group but not in hypertension group; however, age was positively related to t-PSA and IPSS in hypertension group, but it did not correlate with PV in normotension group. Furthermore, t-PSA was positively correlated with PV in hypertension group. These results indicated that there might be no significant association between hypertension and PV. However, further researches with larger sample sizes are warranted to clarify the association between hypertension and BPH.

## Author Contributions

X-TZ, HW, and X-HW designed this study. HW, JX, and L-LM collected data. Y-HJ and L-LM re-checked data. HW and QH performed analysis. X-TZ wrote the manuscript. X-HW reviewed the manuscript.

## Conflict of Interest Statement

The authors declare that the research was conducted in the absence of any commercial or financial relationships that could be construed as a potential conflict of interest.
